# Conference Report: 50th CALORIMETRY CONFERENCE Gaithersburg, MD July 23–28, 1995

**DOI:** 10.6028/jres.101.008

**Published:** 1996

**Authors:** Eugene S. Domalski, Robert N. Goldberg, Patrick A. G. O’Hare

**Affiliations:** Chemical Science and Technology Laboratory, National Institute of Standards and Technology, Gaithersburg, MD 20899-0001

## 1. Introduction

The 50th Calorimetry Conference was held at the National Institute of Standards and Technology during the week July 23–28, 1995. The purpose of the Calorimetry Conference is to improve calorimetric methods and related scientific procedures and to advance their application in science and technology. Towards this end, a Calorimetry Conference is held annually. This year there were 240 registered participants from 22 countries. There were 175 oral and 32 poster presentations. To mark the 50th anniversary of the conference, there was also a special symposium “History of the Calorimetry Conference.” Consistent with the recent history of the conference, the largest number of presentations were in the biochemical and biological areas.

The Calorimetry Conference has always maintained an informal atmosphere and, in this tradition, there are no published proceedings. The principal organizers of the Conference were the Chair, Dr. Eugene S. Domalski (NIST), the Program Chair, Dr. Edwin A. Lewis (Calorimetry Sciences Corp., Provo, Utah), and the Local Arrangements Chair, Dr. Robert N. Goldberg (NIST). Welcoming remarks were presented by the NIST Director, Dr. Arati Prabhakar.

## 2. Conference Summary

Calorimetry and thermodynamics are used in a wide variety of basic and applied sciences. This was reflected in the seemingly disparate topics dealt with at the conference: low- and high-temperature physics, inorganic and organic chemistry, solution chemistry, chemical engineering, biochemistry, and biology. Accordingly, the conference was organized into 11 symposia covering a variety of topics (see Table 1).

## 3. Award Lectures

The Calorimetry Conference presents three awards each year to individual investigators for their work in calorimetry and related areas. It also sponsors the Giauque awards which help students to attend the conference. These latter awards are named in honor of the Nobel laureate whose pioneering work in low-temperature physics led to adiabatic demagnetization and also helped to establish the third law of thermodynamics.

The most prestigious Calorimetry Conference award is the Hugh M. Huffman Memorial Award. Huffman made many substantive contributions to experimental calorimetry and to thermodynamics and was also the prime mover in the establishment of the Calorimetry Conference. This year’s Huffman Award was presented to Dr. Kenneth Breslauer of Rutgers University who presented a lecture titled “DNA Stability and Drug-DNA Interactions: A Thermodynamic Perspective.” A principal theme of his lecture was the correlation of the standard molar Gibbs energy change $\Delta_{r}G_{m}^{{}^{\circ}}$, standard molar enthalpy change $\Delta_{r}H_{m}^{{}^{\circ}}$, and standard molar entropy change $\Delta_{r}S_{m}^{{}^{\circ}}$ with the structural features in DNA. Of particular interest was Dr. Breslauer’s scheme for estimating these thermodynamic quantities for the thermal denaturation of model DNA complexes. Dr. Breslauer also discussed the important role that (drug + DNA) binding studies could play in rational drug design.

The Stig Sunner Memorial Award is presented to a young scientist in recognition of research and other contributions to thermochemistry and thermodynamics. Sunner, the founder of the Thermochemistry Laboratory in Lund, Sweden, made many valuable contributions to combustion and solution calorimetry and to thermochemistry in general. He was also very active in international organizations (IUPAC and CODATA) and worked hard for international cooperation. This year’s Sunner award was presented to Dr. Kenneth P. Murphy of the University of Iowa who presented a lecture titled “Protein Energetics: From Modeled Compounds to Modeled Proteins.” Dr. Murphy emphasized the important relationship between structure, function, and thermodynamics. Most of his lecture was devoted to results obtained by isothermal titration calorimetry on compounds such as cyclic dipeptides. These compounds serve as models for the thermodynamics of protein unfolding and (protein + protein) interactions. The measured quantities $\Delta_{r}H_{m}^{{}^{\circ}}$, $\Delta_{r}S_{m}^{{}^{\circ}}$, and $\Delta_{r}C_{p,m}^{{}^{\circ}}$, the standard molar heat-capacity change, were discussed in terms of both hydrophobic effects and hydrogen bonding. Since interactions of these substances with the solvent are very important, the model described by Dr. Murphy also contained a term for the accessible surface area. This model, while shown to be useful for the estimation of enthalpies of denaturation of globular proteins, did not work well for the estimation of denaturation temperatures.

The James J. Christensen Memorial Award is presented for outstanding contributions to the innovative development and/or use of calorimetric equipment. Christensen was a pioneer in the development of calorimetric methods and apparatus. He also produced a large amount of valuable thermochemical information as well as several very useful compilations of thermodynamic quantities. This year’s Christensen award was presented to Dr. Thomas C. Hofelich of Dow Chemical Co., Midland, Michigan who presented a lecture titled “Calorimetric Instrumentation in Industry: Past Successes, Future Challenges.” Dr. Hofelich reviewed how calorimetry is used in Dow’s Analytical Sciences Laboratory for analysis, for compatibility testing, for shelf-life studies, and for the prediction of thermal runaways. The latter is of considerable industrial importance since a thermal runaway of a chemical in a truck or railroad car can cause serious losses of both property and life. Currently, computer simulation calculations that rely upon the results of calorimetric experiments are used to predict the possibility of thermal runaways. Dr. Hofelich also identified the need for faster and more automated systems, on-line methods of analysis, and reliable reference data and standards.

## 4. History of the Calorimetry Conference

The Symposium on the History of the Calorimetry Conference opened the technical session on the first day of the Conference. This Symposium was an important part of this Conference because after 50 years a historic and significant milestone had been reached. Many trends, innovations, and directions in calorimetric research are reflected in 50 years of presented papers and it was deemed worthwhile to step back and re-examine the past. Hence, in order to emphasize the historic meaning of this Conference, plenary lectures from this Symposium began technical sessions on the first and last days. As part of the focus on 50 years of the Calorimetry Conference, a reprint of a paper written by R. M. Izatt, P. R. Brown, and J. L. Oscarson, and entitled: “The History of the Calorimetry Conference: 1946–1995” [1] was provided to each attendee.

Reed M. Izatt (Brigham Young University, Provo, Utah) presented the first of three Symposium lectures and gave an overview of the history of the Calorimetry Conference. He described the evolution of the Conference from the first meeting in 1946 of a group of low-temperature calorimetrists whose common interest was evaluating problems with discrepant experimental data and improving calorimetric techniques. Through the years, the Calorimetry Conference has evolved into an organization with by-laws and procedures clearly identifying how the annual tasks, traditions, and overall continuity should be passed from one set of elected officers to another.

Izatt discussed the evolution of topics in calorimetry over the past 50 years. A definite shift from low-temperature heat-capacity calorimetry and combustion calorimetry towards solution calorimetry and the calorimetry of biologically important systems is apparent. In conjunction with this trend, one could also see the change from most calorimeters being constructed by the experimentalist to the use of commercial calorimetric equipment.

Special symposia and sessions began to appear in Conference programs in the late 1960s and 1970s. During the 1970s, Conference organizers began to divide papers into named sessions and symposia. These symposia have provided a mechanism not only for topics of common interest to be discussed, but also a means to have invited speakers who are not usual attendees at the Conference present papers on their calorimetric or thermochemical studies. From time to time, joint meetings have been held with organizations such as the North American Thermal Analysis Society (NATAS) and the International Union of Pure and Applied Chemistry (IUPAC), and served as a means for technology transfer and information exchange between scientists. Izatt also reviewed the three major Conference awards (Huffman, Sunner, and Christensen) given in recognition of outstanding achievements in calorimetry and the Giauque Award to aid students to attend the Conference and to encourage them to present papers on their research.

The second speaker of the Symposium, Daniel R. Stull (retired, Dow Chemical Co., Midland, Michigan) spoke on “The Early Years of the Calorimetry Conference.” Stull was very active in the early Calorimetry Conferences and has written excellent histories on its early years [2,3]. He recalled that in the late 1940s it was a matter of concern that the thermodynamic properties of isopentane measured in various low-temperature heat-capacity laboratories showed pronounced disagreement. Hugh M. Huffman sent letters to the calorimetry community in the spring and early summer of 1946, and suggested that an informal conference be held of persons interested in low-temperature calorimetry. The agenda for discussion by participants included the following topics: temperature scales, time measurements, electrical measurements and instrumentation, shielding and other protection from electrical leaks, design of calorimeters and cryostats, heat interchange between calorimeter and environment, mass determination, methods of calculation, and standard test substances. The primary purpose of the informal conference was to discuss the calorimetric methods with particular emphasis on their effect on the final accuracy of the measurements. From such discussions, the problems of disparity in the thermal data for isopentane could be examined and evaluated. Huffman served as chairman for the first three group meetings in 1946, 1947, and 1948. The fourth meeting of the low-temperature calorimetrists was chaired by Stull at the Massachusetts Institute of Technology in Cambridge, Massachusetts in September 1949. Huffman was absent from this meeting because he needed to attend the first IUPAC General Assembly meeting in Amsterdam which took place during the same time period. Hugh Huffman’s unexpected and untimely death in January of 1950 at the age of 51 came as a shock to the low temperature calorimetry community. The fifth group meeting was held at Northwestern University in Evanston, Illinois in 1950 and was designated as the “Hugh M. Huffman Memorial Meeting.” The meeting was chaired by Stull who also chaired the next two meetings. The meeting at Northwestern University was the first time that the group used the name “Calorimetry Conference.” As meetings of the low-temperature calorimetrists took place over the next several years, much progress was made regarding the calibration of platinum resistance thermometers, selection of standard substances for the calibration of low-temperature heat-capacity calorimeters, and the best choice for auxiliary instrumentation.

The third speaker, Gus Somsen (Vrije Universiteit, Amsterdam, The Netherlands), gave a presentation on “The International Ties of the Calorimetry Conference.” He pointed out that Hugh M. Huffman himself attended the first IUPAC General Assembly meeting after World War II. This meeting was held in Amsterdam in 1949. The 16th Calorimetry Conference was held in Ottawa, Canada as a joint meeting with the IUPAC Subcommittee on Experimental Thermochemistry and was called the “1961 International Calorimetry Conference.” In 1971, a joint IUPAC Conference was held with the 26th Calorimetry Conference in Orono, Maine. Joint Calorimetry Conferences were held with the 8th IUPAC Conference in 1984 in Hamilton, Ontario, and with the 12th IUPAC Conference in 1992 in Snowbird, Utah. Except for the period from 1979 to 1984, at least one of the members of the Calorimetry Conference’s Board of Directors was from overseas. Also, seven of the Huffman Memorial Award recipients have come from Europe or Asia.

The morning of the last day of the conference saw five speakers conclude the Symposium on the History of the Calorimetry Conference.

Ingemar Wadsö (University of Lund, Lund, Sweden) spoke on “Developments of Calorimetry at Lund and its Link with the Calorimetry Conference.” The initial ties between the Thermochemical Laboratory at Lund University and the Calorimetry Conference were formed when Stig Sunner spent some time at the Thermodynamics Laboratory in Bartlesville in the 1950s. At that time both laboratories actively pursued the bomb calorimetry of organic sulfur compounds. Many aspects of the research effort were shared and pursued together, such as bomb rotation, the attainment of sample purity, combustion of common samples, establishment of the final state of sulfur as a uniform sulfuric acid solution, and corrections of the calorimetric data to the standard state. During the 1960s, both the Thermochemical Laboratory at the University of Lund and the Thermochemical Laboratory at Brigham Young University in Provo were pursuing joint research in solution and reaction calorimetry. Also, studies on biochemical systems and in cell biology using microcalorimetric apparatus at Lund broadened its contact with North American calorimetrists.

Eugene S. Domalski (NIST, Gaithersburg, Maryland) gave a presentation on “NIST/NBS Involvement in 50 Years of the Calorimetry Conference.” Brief curriculum vitae and research highlights were provided for past NIST/NBS Huffman Memorial Awardees. The awardees were Ferdinand G. Brickwedde, Frederick D. Rossini, Charles W. Beckett, Edward J. Prosen, and Patrick A. G. O’Hare. Brickwedde, Rossini, and Prosen were active in the early years, 1946–1955. Rossini received the Huffman Award after leaving NBS for the Carnegie Institute of Technology. Beckett and Prosen again were active during 1955–1980, and O’Hare has been active since the late 1960s and received the Huffman Award prior to coming to NIST. Research areas discussed included temperature scales in relation to calorimetry, chemical thermodynamics in IUPAC, thermochemistry of hydrocarbons, progress in thermodynamics and related R&D, and the thermodynamics of inorganic chalcogenides. Other NIST/NBS staff members (George T. Armstrong, Jane E. Callanan, Kenneth L. Churney, Jennifer C. Colbert, Eugene S. Domalski, George T. Furukawa, Robert N. Goldberg, Duane R. Kirklin, Yadu B. Tewari, and E. Dale West) have served as officers or on the Conference’s Board of Directors.

J. Bevan Ott (Brigham Young University, Provo, Utah) presented an overview of “Forty Years of Calorimetry at Brigham Young University (BYU).” In his talk, he traced the development of calorimetry at BYU since 1955 and summarized some of the major work accomplished by various investigators. Of particular importance were the early contributions of James J. Christensen and Reed M. Izatt in the development of titration calorimetry and its use in acquiring a remarkable amount of thermochemical information. These and other workers at BYU always maintained close ties to the Calorimetry Conference. This was reflected in the large number of staff members (Juliana Boerio-Goates, Delbert J. Eatough, Reed M. Izatt, John L. Oscarson, J. Bevan Ott, Jadwiga T. Sipowska, Earl M. Woolley) who served as either officers or on the Board of Directors of the Calorimetry Conference and who received several of its prestigious awards (James J. Christensen, Reed M. Izatt, J. Bevan Ott, Delbert J. Eatough, Juliana Boerio-Goates, and Lee D. Hansen). Also, Calorimetry Conferences were hosted by BYU in 1972, 1982, and 1992. Ott summarized some of the recent instrumental advances and applications of calorimetry at BYU in the measurement of enthalpies of mixing and heat capacities, the study of biochemical and biological systems, and high temperature chemistry. He credited many of the advances in these areas to the cross fertilization of ideas that had come about during Calorimetry Conferences.

Gerald K. Johnson (Argonne National Laboratory, Argonne, Illinois) gave a lecture on “The Calorimetry Conference and Argonne National Laboratory, Fifty Years of Collaboration” in which he highlighted several calorimetric programs that, at one time or another, had been in progress at Argonne National Laboratory (ANL). Those included: fluorine combustion calorimetry and solution calorimetry (Ward N. Hubbard); low-temperature calorimetry (Darrell W. Osborne and Howard E. Flotow); synthesis calorimetry (Yehuda Baskin); and solution calorimetry in molten metals (Joseph B. Darby, Jr.). Johnson also pointed out that several scientists from ANL had been intimately involved with the Conference as either Chairman or Director, or as providers of reference samples (e.g., copper for low-temperature calorimetry). Over the years, ANL supplied four winners of the Huffman Memorial Award (W. N. Hubbard, D. W. Osborne, H. E. Flotow, and P. A. G. O’Hare).

William V. Steele (NIPER, Bartlesville, Oklahoma) spoke about “Bartlesville and the Calorimetry Conference.” The Thermodynamics Laboratory in Bartlesville shares all of the history of the 50 years of the Calorimetry Conference because Hugh M. Huffman was the founder of both the Laboratory and the Conference. The pursuit of accurate thermodynamic properties of hydrocarbons was an important research area in the 1940s and 1950s. The calorimetric work carried out on organic sulfur compounds in the 1950s and 1960s came as a consequence of their presence in hydrocarbons as an impurity. Their characterization resulted in the development of appropriate extraction and separation techniques. During the 1970s, 1980s, and 1990s, the intermittent research on coal and coal-derived liquids as alternative fuels gave impetus to thermodynamic measurements on a large number of organic nitrogen compounds at Bartlesville. Four calorimetrists from the Bartlesville Laboratory were recipients of the Huffman Memorial Award (Guy Waddington, John P. McCullough, William D. Good, and William V. Steele). Robert D. Chirico was a Sunner Award recipient.

## 5. Thermodynamics and Industry

The symbiotic relationship between users and generators of thermodynamic information was the dominant theme of this symposium.

Applications of thermodynamics in the specialty chemicals industry was the focus of several lectures that dealt with such topics as safety and environmental concerns, simulation of multicomponent mixtures, phase equilibria, development of chemical models for systems at extremes of temperature and pressure, and prediction of properties considered too costly or too time-consuming to be determined experimentally. The premium placed on reliable, critically evaluated databases used in such applications was also emphasized. Particular calorimetric techniques developed to address specific problems were discussed. In that connection, the problem of shelf life of chemicals figured largely. The continuing demand for reliable experimental thermodynamic quantities also formed a recurrent motif.

The prominence of metal silicides in the “high-tech” industries was reflected by the large number of papers that dealt with the thermodynamic behavior of such materials. Featured here were measurements over wide ranges of temperature on titanium, cobalt, and molybdenum silicides, both as bulk materials and thin films. Many other substances used in modern technologies were discussed. They included NiZr_2_, Pd/Sn diffusion couples, and superconducting oxides. Accurate thermodynamic information was reported for the xylenes and their equilibrium mixtures. A mercury thermostat was described for use with platinum resistance thermometers. In the environmental area, papers were presented that dealt with the application of thermodynamics to such diverse topics as spontaneous combustion of low-rank coals, and the reprocessing of spent sulfuric acid from the manufacture of titanium dioxide.

## 6. Panel Discussion on “The Need for Standards in Calorimetry”

During the early years of the Calorimetry Conference, an effort was begun to develop standard samples for calibration purposes which were to be used in low temperature heat capacity measurements. It was felt that comparative measurements among calorimetric laboratories with the same standard substances should help to resolve problems with discrepant experimental data. Also, the interchange of data resulting from the use of such samples was encouraged. From these efforts, several low-tempertature heat-capacity calorimetry standards were developed and were made available through the years for distribution to the Conference membership from NIST/NBS. The samples were sapphire (alumina), benzoic acid, copper, and n-heptane.

With a variety of in-house constructed or modified calorimeters as well as commercial calorimeters currently being used by researchers, concern has grown with respect to the proper choice of standard samples and to the use of acceptable standard procedures for calibration. Possible standard samples or standard reactions were discussed during the panel meeting for different types of calorimetry such as titration calorimetry and microcalorimetry. No definite consensus was reached regarding candidate samples or reactions. It was decided that further thought and study were needed. Thomas C. Hofelich was appointed as chairman of the Committee on Standard Samples with the goal of renewing discussions on this topic at the next Calorimetry Conference in Vancouver, British Columbia in August 1996.

## 7. The Next Conference

The 51st Calorimetry Conference will be held August 4–9, 1996 at The University of British Columbia, Vancouver, British Columbia. Information on the program can be obtained from the Program Chair, Dr. William V. Steele, National Institute for Petroleum and Energy Research, Bartlesville, OK 74005. Local Arrangements information can be obtained from Dr. Yoshikata Koga, Department of Chemistry, The University of British Columbia, Vancouver, British Columbia V6T 1Z1, Canada.

## Figures and Tables

**Table 1:** Symposia of the 50th Calorimetry Conference

**The History of the Calorimetry Conference.** Chair: Dr. Robert N. Goldberg (NIST).
Plenary Lecture: “The Calorimetry Conference: 1946 to 1995,” Dr. Reed M. Izatt, (Brigham Young University).
Invited Lectures by: Dr. Daniel R. Stull (Dow Chemical Co., retired), Dr. Gus Somsen (Vrije Universiteit, The Netherlands), Dr. Ingemar Wadsö (Lund University, Sweden), Dr. Eugene S. Domalski (NIST), Dr. J. Bevan Ott (Brigham Young University), Mr. Gerald Johnson (Argonne National Laboratory), and Dr. William Steele (National Institute for Petroleum and Energy Research).
**Stability and Binding of Macromolecules.** Chair: Dr. Kenneth P. Murphy (University of Iowa).
Plenary Lecture: “Thermodynamics of Protein Stability and Molecular Recognition,” Dr. Ernesto Freire (The Johns Hopkins University).
Invited Lecture by: Dr. Michael L. Doyle (Smith-Kline Beecham Pharmaceuticals).
**Thermodynamics of Model Systems Related to the Binding and Assembly of Biological Macromolecules.** Chair: Dr. Patrick Connelly (Vertex Pharmaceuticals, Inc.).
Plenary Lecture: “Contributions of Hydrogen Bonding in the Hydrophobic Effect to Protein Stability,” Dr. C. Nick Pace (Texas A&M University).
Invited Lectures by: Dr. Julian Sturtevant (Yale University), Dr. Phil Ross (National Institutes of Health), Dr. George Makhatadze (The Johns Hopkins University), and Dr. John Ladbury (Oxford University, U.K.).
**Methods in Biothermodynamics.** Chair: Dr. Fred Schwarz (NIST).
Plenary Lecture: “New Developments in Microcalorimetry,” Dr. Peter L. Privalov (The Johns Hopkins University).
Invited Lectures by: Dr. Avedhesha Surolia (Indian Institute of Science, India), Dr. Andrew Shrake (Food and Drug Administration), and Dr. Robert N. Goldberg (NIST).
**Thermodynamics and Industry.** Chair: Dr. P. A. G. O’Hare (NIST).
Plenary Lecture: “What’s Hot and What’s Not: Thermodynamics in the Organic Chemical Industry,” Dr. Dennis R. Cordray (Union Carbide Corp.).
Invited Lectures by: Dr. Gregory A. Hope (Griffith University, Australia), Dr. J. I. Macnab (Zeneca, U.K.), Dr. David J. Frurip (Dow Chemical Co.), and Dr. Eric J. Cotts (Binghamton University).
**Calorimetry of Nuclear Materials.** Chair: Mr. Lee Refalo (Westinghouse Savannah River Co.).
Plenary Lecture: “Calorimetry at the Savannah River Site,” Mr. Henry Randolph (Westinghouse Savannah River Co.).
Invited Lecture by: Dr. M. Fred Duff (Mound EG&G Applied Technologies).
**Advances in Calorimetric Instrumentation.** Chair: Dr. Lee Hansen (Brigham Young University).
Invited Lecture by: Dr. Stanislaw L. Randzio (Polish Academy of Sciences, Poland).
**Enthalpies of Mixing of Non-electrolytes.** Chair: Dr. Phillip Brown (Brigham Young University).
Plenary Lecture: “Enthalpies of Mixing of Non-electrolytes—25 Years of Advances in Measurement Techniques,” Dr. Kenneth N. Marsh (Texas A&M University).
**Aqueous Electrolyte Solutions.** Chairs: Dr. Joseph Rard (Lawrence Livermore National Laboratory) and Dr. Earl Woolley (Brigham Young University).
Plenary Lecture: “Experimental Investigations of Volumetric and Caloric Properties of Aqueous Solutions at High Temperature and Pressure,” Dr. Vladimír Majer (Université Blaise Pascal, France).
Invited Lectures by: Dr. Peter Tremaine (Memorial University of Newfoundland, Canada) and Dr. J. M. Simonson (Oak Ridge National Laboratory).
**Fire Calorimetry.** Chair: Dr. Richard Lyon (Federal Aviation Administration Technical Center).
Plenary Lecture: “Fire Science and Engineering—Why So Hard?,” Dr. John W. Lyons (Army Research Laboratory).
Invited Lectures by: Dr. Richard G. Gann (NIST), Dr. Marc L. Janssens (American Forest and Paper Association), Dr. Edwin E. Smith (Ohio State University), Dr. Vytenis Babrauskas (Fire Science and Technology, Inc.), Dr. Archibald Tewarson (Factory Mutual Research Corp.), Dr. Takashi Kashiwagi (NIST), Dr. Arthur Grand (Omega Point Laboratories), Dr. Björn Karlsson (Lund University, Sweden), Dr. Jeffrey S. Newman (Factory Mutual Research Corp.), Dr. Marcelo M. Hirschler (GBH International), and Dr. Richard W. Bukowski (NIST).
**General.** Chair: Dr. Edwin A. Lewis (Calorimetry Sciences Corp.)
Plenary Lectures: “Molecular Motion, Lattice Vibration, and Phase Transition Phenomena in C_60_ and C_70_ and Their Compounds,” Dr. Tooru Atake (Tokyo Institute of Technology, Japan) and “Towards a Global Equation of State for Protein Unfolding,” Dr. Harold C. Helgeson (University of California, Berkeley).
Invited lecture by: Dr. Victor P. Kolesov (Moscow State University, Russia).
